# Micromotor‐Enabled Active Hydrogen and Tobramycin Delivery for Synergistic Sepsis Therapy

**DOI:** 10.1002/advs.202303759

**Published:** 2023-10-11

**Authors:** Yanzhen Song, Ruotian Zhang, Hanfeng Qin, Wenxin Xu, Jia Sun, Jiamiao Jiang, Yicheng Ye, Junbin Gao, Huaan Li, Weichang Huang, Kun Liu, Yunrui Hu, Fei Peng, Yingfeng Tu

**Affiliations:** ^1^ NMPA Key Laboratory for Research and Evaluation of Drug Metabolism & Guangdong Provincial Key Laboratory of New Drug Screening School of Pharmaceutical Sciences Southern Medical University Guangzhou 510515 China; ^2^ School of Materials Science and Engineering Sun Yat‐Sen University Guangzhou 510275 China

**Keywords:** drug delivery, hydrogen, micromotor, sepsis, synergistic therapy

## Abstract

Sepsis is a highly heterogeneous syndrome normally characterized by bacterial infection and dysregulated systemic inflammatory response that leads to multiple organ failure and death. Single anti‐inflammation or anti‐infection treatment exhibits limited survival benefit for severe cases. Here a biodegradable tobramycin‐loaded magnesium micromotor (Mg‐Tob motor) is successfully developed as a potential hydrogen generator and active antibiotic deliverer for synergistic therapy of sepsis. The peritoneal fluid of septic mouse provides an applicable space for Mg‐water reaction. Hydrogen generated sustainably and controllably from the motor interface propels the motion to achieve active drug delivery along with attenuating hyperinflammation. The developed Mg‐Tob motor demonstrates efficient protection from anti‐inflammatory and antibacterial activity both in vitro and in vivo. Importantly, it prevents multiple organ failure and significantly improves the survival rate up to 87.5% in a high‐grade sepsis model with no survival, whereas only about half of mice survive with the individual therapies. This micromotor displays the superior therapeutic effect of synergistic hydrogen‐chemical therapy against sepsis, thus holding great promise to be an innovative and translational drug delivery system to treat sepsis or other inflammation‐related diseases in the near future.

## Graphical abstract

The peritoneal fluid of septic mice activates the Mg‐water reaction for the in‐situ production of hydrogen. Hydrogen generated sustainably and controllably from the motor interface functions not only as a propellant for the motion, but also as an active ingredient for inflammation scavenging. Meanwhile, the continuous detachment of hydrogen also results in active tobramycin delivery that allows for enhanced antibiotic therapy.

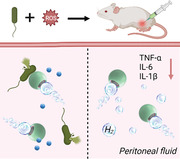

## 1 Introduction

Sepsis is a life‐threatening highly heterogeneous syndrome attributed to the dysregulated host response to bacterial infection, with high mortality.^[^ ^1^ ^]^ The current treatments, including antibiotic administration, fluid resuscitation and hemodynamic support,^[^ ^2^ ^]^ exhibit limited survival benefit for severe cases. It is generally considered that bacterial infection only acts as a trigger for sepsis, and the early death is probably predominantly caused by postinfectious hyperinflammation.^[^ ^3^ ^]^ Unfortunately, current therapeutic strategies to mitigate overwhelming inflammation and its complications in sepsis remain insufficient.

Although the jury is still out on sepsis pathogenesis, it has been documented that lipopolysaccharide (LPS) on the outer membrane of Gram‐negative bacteria can bind to Toll‐like receptors on immune cells and then induce a large release of inflammatory cytokines, triggering cytokine storm. If these cytokines are not cleared in time, which will cause irreversible damage to epithelial, endothelial and immune cells, eventually leading to multiple organ failure and death.^[^ ^4^ ^]^ However, therapies developed to eliminate individual cytokine such as tumor necrosis factor‐alpha (TNF‐α)^[^ ^5^ ^]^ and interleukin‐1β (IL‐1β)^[^ ^6^ ^]^ have failed to raise the survival rate. Sepsis obviously manifests itself in a heterogeneous inflammation response, and single‐target therapies are inadequate for severe cases. Hence, we believe that developing a new therapeutic approach capable of scavenging multiple inflammatory mediators, combining with moderate antibiotic administration, is of great importance to suppress systemic inflammation and improve the survival rate of high‐grade sepsis.

Over the past decade, antioxidant therapy has emerged as a novel strategy to mitigate inflammation in reactive oxygen species (ROS)‐related diseases, including sepsis. Remarkably, WS_2_ nanosheets^[^ ^7^ ^]^ and ceria‐zirconia nanoparticles^[^ ^8^ ^]^ as enhanced multiple ROS scavenger have shown promise in alleviating overwhelming inflammation and its complications of sepsis. In the meantime, hydrogen (H_2_) has also been spotlighted as a potential antioxidant due to its selective removal of cytotoxic oxygen radicals^[^ ^9^ ^]^ and activation of endogenous antioxidant enzymes.^[^ ^10^ ^]^ Furthermore, its ability to effectively counteract a range of pro‐inflammatory cytokines, such as TNF‐α, IL‐6 and IL‐1β, has been confirmed, possibly through the suppression of nuclear factor‐κB, which is a pro‐inflammatory transcription factor.^[^ ^11^ ^]^ Recently, the utilization of H_2_ for sepsis therapy has gained significant popularity owing to its survival benefit derived from multicomponent anti‐inflammatory activity, as well as other biological effects encompassing anti‐apoptosis, anti‐shock and autophagy regulation.^[^ ^12^ ^]^ Xie et al. demonstrated that H_2_ inhalation significantly alleviated multiple organ damage and enhanced the survival rate of septic mice.^[^ ^13^ ^]^ Nonetheless, the inherent limitations of H_2_, namely its short life span and low solubility, pose challenges to achieving sustainable and controlled administration through passive delivery system, thereby potentially compromising the therapeutic efficacy.

Micro/nanomotors (MNMs) that capable of converting physical or chemical energies into mechanical motion may offer the possibility to solve the above‐mentioned challenges in H_2_ therapy. MNMs have been applied for tremendous biomedical applications ^[^ ^14^ ^]^ involving micro‐/nano surgery,^[^ ^15^ ^]^ assisted fertilization,^[^ ^16^ ^]^ environmental remediation ^[^ ^17^ ^]^ and targeted drug delivery.^[^ ^18^ ^]^ Notably, these MNMs show great potential for drug delivery due to their active propulsion capability along with efficient drug loading and enhanced penetration into deep tissues.^[^ ^19^ ^]^ In the case of chemical propelled MNMs, hydrogen peroxide, as a commonly used fuel to drive the motion is toxic, potentially impeding their suitability for biomedical applications. This has led to the exploration of alternative, safe fuel sources, such as employing enzymes as biocatalytic units for propulsive power.^[^ ^20^ ^]^ At the same time, magnesium‐based (Mg‐based) micromotors have also attracted increasing research interest for their active propulsion solely relied on spontaneous reaction between Mg and acid‐ or water‐based biofluids.^[^ ^21^ ^]^ Furthermore, based on previous studies, Mg‐based micromotors have been successfully employed for H_2_ therapy^[^ ^22^ ^]^ and active drug delivery,^[^ ^23^ ^]^ owing to their sustained and controlled H_2_ production capacity as well as inherent mobility. Considering the promising outcomes associated with each of these advantages, we firmly believe that integrating the two aspects would hold great potential for the treatment of complex diseases.

**Figure 1:**
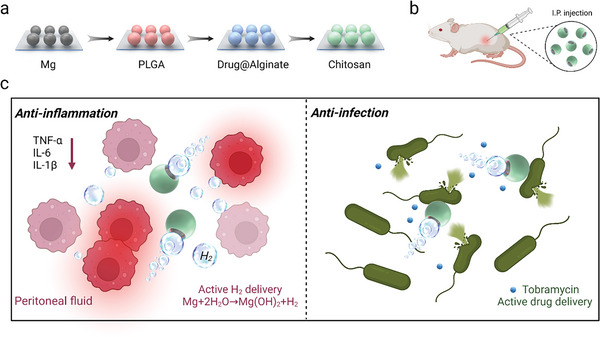
Schematic illustration of the fabrication and in vivo application of Mg‐Tob motors. a) Schematic preparation steps. Mg‐Tob motors are fabricated by asymmetrically coating Mg microparticles with a PLGA layer following by tobramycin‐loaded sodium alginate and chitosan coatings. b) Intraperitoneal injection of Mg‐Tob motors. c) Synergistic hydrogen‐chemical therapy against sepsis. As a potential H_2_ generator and active antibiotic deliverer, Mg‐Tob motors exhibit dual therapeutic effect of anti‐inflammation as well as anti‐infection.

Herein, we demonstrate for the first attempt to apply Mg‐based micromotors, loaded with antibiotic tobramycin, for in vivo combined treatment of sepsis in a cecal ligation and puncture (CLP) mouse model (**Figure** **1**). Mg‐Tob motors are fabricated by asymmetrically coating Mg microparticles with a biodegradable polylactic acid‐glycolic acid copolymer (PLGA) layer following by a tobramycin‐loaded alginate‐chitosan hydrogel. The peritoneal fluid of septic mouse provides an applicable space for Mg‐water reaction. Hydrogen generated sustainably and controllably from the motor interface functions as not only a propellant for the motion, but also an active ingredient for inflammation scavenging. Meanwhile, the continuous detachment of the produced hydrogen also results in active tobramycin delivery, thus exhibiting dual therapeutic effect of anti‐inflammation and anti‐infection. As expected, positively charged chitosan coating only exhibits weak antimicrobial activity. By combining with the antibiotic, our Mg‐Tob motors demonstrate reduced organ damage as well as significantly improved survival rate up to 87.5% in a high‐grade sepsis model with no survival. Together, our micromotors display an attractive therapeutic effect of synergistic hydrogen‐chemical therapy against sepsis, which may shed a light on the effective treatment of sepsis and other inflammation‐related diseases in the near future.

## 2 Results and discussion

### 2.1 Fabrication and Characterization of Mg‐Tob motors

**Figure 2:**
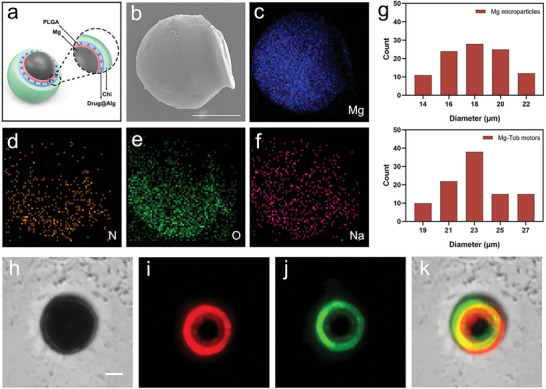
Characterization of Mg‐Tob motor. a) Schematic illustration of the multilayer structure of Mg‐Tob motor. b) SEM image of a Mg‐Tob motor. Scale bar: 10 µm. c‐f) Element mapping of Mg, N, O, Na. g) Size distribution of Mg microparticles and Mg‐Tob motors (n = 100). h‐k) Bright‐field and fluorescence images of a Mg‐Tob motor with Nile red and FITC labeling. Scale bar: 10 µm.

**Figure** **2**a schematically illustrates the multilayer structure of Mg‐Tob motor. In the fabrication process, Mg microparticles were first dispersed over a glass slide pre‐covered with polyvinyl pyrrolidone (PVP) film, then the biodegradable polymers, PLGA, sodium alginate (Alg) containing antibiotic tobramycin (Tob) and chitosan (Chi) were sequentially coated onto Mg microparticles, allowing a small opening for spontaneous Mg‐water reaction in peritoneal fluid. PLGA was used to prevent the formation of a MgO passive layer and to ensure the stability of motors during the preparation. Negatively charged Alg and polycationic Chi could form a hydrogel structure through electrostatic interaction.^[^ ^24^ ^]^ Scanning electron microscope (SEM) image of a Mg‐Tob motor (Figure 2b) demonstrated the presence of a small opening. Meanwhile, energy‐dispersive X‐ray spectroscopy (EDX) was performed to fully characterize the element composition of Mg‐Tob motor. The distribution of elements involving Mg from Mg microparticles, N from Chi, Na from Alg and common element (O) were clearly observed (Figure 2c–f). As shown in Figure 2g, Mg‐Tob motor exhibited a larger size (∼23 µm diameter) than that of Mg microparticle (∼18 µm diameter, Figure S1). The formed PLGA layer and Alg‐Chi hydrogel were then labeled with hydrophobic Nile red and hydrophilic fluorescein isothiocyanate (FITC) respectively, further indicating the multilayer structure of Mg‐Tob motor (Figure 2h–k).

### 2.2 Drug and Hydrogen Release from Mg‐Tob motors

After confirming the successful fabrication of Mg‐Tob motor, fluorescence spectrophotometry was further used to quantify the Tob loading content.^[^ ^25^ ^]^ Based on the calibration curve (Figure S2), the loading capacity of Tob was around 51.72 ± 0.69 µg per 2.5 mg Mg‐Tob motors. Due to the small opening of Mg‐Tob motor, the uncovered Mg could react with water gradually, resulting in sustainable and controllable generation of H_2__._ H_2_ detection is based on the Redox reaction between methylene blue (MB) and H_2_ under the catalysis of Pt nanoparticles. The former is able to form colorless reduced leucomethylene blue (leucoMB) to quantify the H_2_ release by calculating the absorbance difference of sample at 664 nm according to the constructed calibration curve (Figure S3). As shown in Figure S4, Mg microparticles exhibited an initial burst release of H_2_, which was reflected in the significantly decreased MB absorbance in the first 10 mins. Whereas for Mg‐Tob motors and Mg‐Tob microparticles, MB absorbance declined gradually in 60 mins, illustrating the sustained release of H_2_. With this controlled and sustained H_2_ release capability, our Mg‐Tob motors are capable of efficient movement and are suitable for H_2_ therapy. Additionally, due to their highly efficient motion, Mg‐Tob motors demonstrated rapid Tob release behavior (Figure S5).

### 2.3 Motion of Mg‐based motors

**Figure 3:**
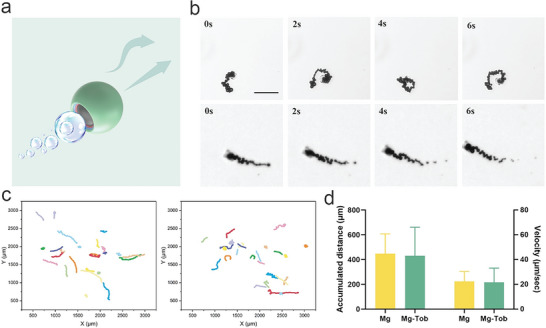
Motion of Mg‐based motors. a) Two moving patterns of Mg‐based motors. b) Spiral and linear motion of motors in MPF. Scale bar: 100 µm. c) Optical tracking trajectories (20 s) of Mg motors and Mg‐Tob motors in MPF respectively. d) Accumulated distances and velocities of Mg motors and Mg‐Tob motors in MPF respectively (n = 25).

Motion behaviors of both Mg motors (without loading Tob) and Mg‐Tob motors under mouse peritoneal lavage fluid (MPF) were then evaluated by a Nikon Ti2‐A inverted optical microscope. MPF was acquired by collecting the peritoneal lavage fluid of septic mouse after injecting intraperitoneally 3 mL of PBS. Schematic of two moving patterns was shown in **Figure** **3**a, and spiral and linear motions were observed in MPF (Figure 3b, captured from Videos S1 and S2). Using software Image J and Chemotaxis, the trajectory (Figure 3c), accumulated distance and velocity (Figure 3d) of motors were obtained. It is worth mentioning that no apparent velocity difference was observed between Mg motors (22.45 ± 7.92 µm ^−1^s) and Mg‐Tob motors (21.59 ± 11.48 µm ^−1^s), suggesting that the antibiotic payload did not compromise the motion capability. The accumulated distance of Mg‐Tob motors was 431.84 ± 229.65 µm, similar to that of Mg motors (448.90 ± 158.43 µm, 20 s). The above results confirmed that the locomotion of our Mg‐Tob motors was highly efficient even in the complex peritoneal fluid environment, which endowed our motors with the capability of active H_2_ and drug delivery. Besides, we have monitored the gradual Mg depletion in MPF. Single Mg‐Tob motor displayed a lifetime of approximately 8 min. As shown in Video S3 and Figure S6, once the Mg core degraded completely due to the Mg‐proton reaction, the motor ceased its movement and only the outer degradable shell including PLGA, Alg and Chi remained.

### 2.4 Biocompatibility and Anti‐Inflammatory Activity of Mg‐Tob motors In Vitro

Murine macrophage cell line, RAW 264.7, is commonly used for inflammation‐related studies. Before evaluating the intracellular inflammation attenuation efficacy of Mg‐Tob motor, its biocompatibility was detected by investigating the cytotoxicity against RAW 264.7 cells. Mg motors and tobramycin‐loaded silicon dioxide microparticles (SiO_2_‐Tob microparticles) with an average diameter of 20 µm were fabricated by the same approach of tobramycin loading, were applied as control groups. The two kinds of Mg‐based motors (Mg motors, Mg‐Tob motors) and SiO_2_‐Tob microparticles all showed no significant cytotoxicity, with more than 90% of RAW 264.7 cells remaining viable even at high concentrations after 24 h incubation (Figure S7), indicating the favorable biocompatibility of Mg‐Tob motors as H_2_ generators, which is crucial for in vivo sepsis treatment. Based on the result, 0.4 mg/mL of Mg motors, 1 mg/mL of Mg‐Tob motors, 1.2 mg/mL SiO_2_‐Tob microparticles (containing the same concentration of magnesium and tobramycin respectively, Figure S8) were used in the following experiments.

**Figure 4:**
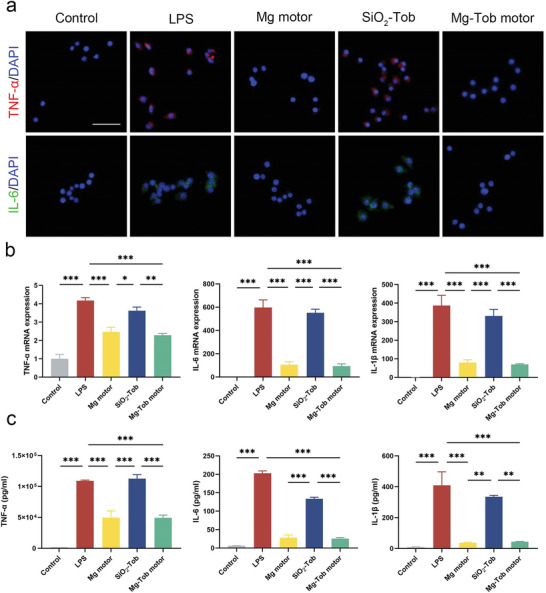
In vitro anti‐inflammatory activity of Mg‐Tob motors. a) Immunofluorescence images of TNF‐α (red) and IL‐6 (green) in LPS‐induced RAW 264.7 cells with different treatments. Scale bar: 50 µm. b) The mRNA expression levels of TNF‐α, IL‐6 and IL‐1β in LPS‐induced RAW 264.7 cells after different treatments. c) The concentrations of TNF‐α, IL‐6 and IL‐1β in LPS‐induced RAW 264.7 cells after different treatments. Data represent mean ± SEM (n = 3), and the significant difference was analyzed by One‐way ANOVA with Tukey's post hoc test. *p < 0.05, **p < 0.01, ***p < 0.001 and ns representing non‐significance.

Mg‐based motors could generate efficient H_2_ during self‐propulsion and neutralize multiple pro‐inflammatory cytokines, thus achieving the intracellular inflammation attenuation. Immunofluorescence staining was then carried out to visualize the intracellular expression of pro‐inflammatory cytokines involving TNF‐α (red) and IL‐6 (green) in LPS‐stimulated RAW 264.7 cells. As shown in **Figure** **4**a, after being stimulated by LPS for 12 h, it was clear that RAW 264.7 cells showed strong red and green fluorescence in addition to blue fluorescence from cell nucleus, illustrating the increased expression of TNF‐α and IL‐6, which are largely associated with inflammation reaction. The groups treated with Mg motors and Mg‐Tob motors displayed a significant drop of these proinflammatory cytokines. Both of them emitted weak or even invisible red and green fluorescence, while the SiO_2_‐Tob microparticles group showed the same result with LPS group. Moreover, the real‐time polymerase chain reaction (Real‐time PCR) and enzyme‐linked immunosorbent assays (ELISA) were further performed to investigate the anti‐inflammatory activity of Mg‐based motors by inhibiting the excessive secretion of acute‐phase inflammatory cytokines (TNF‐α and IL‐1β) and chronic‐phase inflammatory cytokine (IL‐6). After being stimulated by LPS for 12 h, the messenger RNA (mRNA) expression levels of these proinflammatory cytokines increased significantly compared to the negative control group, indicating the successful establishment of cell inflammation model. Relatively lower levels were observed after incubating with Mg motors and Mg‐Tob motors (Figure 4b), which confirmed the strong anti‐inflammatory activity of H_2_ again. Meanwhile, the corresponding concentrations of these proinflammatory cytokines decreased to the lowest level after treated with Mg‐based motors according to ELISA results (Figure 4c). In the above experiments, no statistical difference was seen in the expression levels of inflammatory cytokines between the SiO_2_‐Tob microparticles group and LPS group, further supporting the powerful anti‐inflammatory activity solely acquired from H_2_, a byproduct of Mg‐water reaction. Together, these results suggested that Mg‐Tob motors conveyed satisfactory anti‐inflammation protection by neutralizing multiple pro‐inflammatory cytokines at cell level, which ensured our motor as an excellent candidate to prevent the development of overwhelming inflammation in sepsis.

### 2.5 Survival Analysis and Anti‐Inflammatory Performance In Vivo

**Figure 5:**
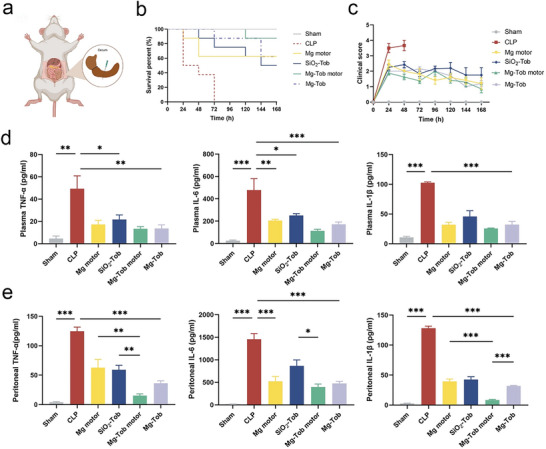
Survival analysis and anti‐inflammatory performance in vivo. a) Schematic of CLP‐induced high‐grade sepsis (75% of cecum was ligated). b) Survival rates of mice after different treatments (n = 8). c) Clinical scores of mice after different treatments during 7 d. d‐e) The concentrations of TNF‐α, IL‐6 and IL‐1β in plasma and peritoneal cavity. Data represent mean ± SEM (n = 5), and the significant difference was analyzed by One‐way ANOVA with Tukey's post hoc test. *p < 0.05, **p < 0.01, ***p < 0.001 and ns representing non‐significance.

In vivo therapeutic effect of Mg‐Tob motors was then evaluated using a mouse sepsis model, which was induced by CLP mimic the pathogenesis and progression of human sepsis.^[^ ^26^ ^]^ As for CLP model, the position of cecal ligation is the decisive factor of severity level and mortality of sepsis. In general, with 75% of cecum ligating, all mice die within 4 d after CLP, which is defined as high‐grade sepsis. Whereas for mid‐grade sepsis, 50% of cecum is ligated, resulting in a survival rate about 40%.^[^ ^27^ ^]^ In our study, we ligated around 75% of the cecum to construct a high‐grade sepsis model with no survival (**Figure** **5**a). We compared the survival rate of Mg‐Tob motors with various control groups including the sham, CLP, Mg motors (single hydrogen therapy), SiO_2_‐Tob microparticles (single drug therapy) and Mg‐Tob microparticles (synergistic hydrogen‐chemical therapy without movement, Video S4) (Figure 5b). Sham mice underwent the same laparotomy except for CLP, in which there were no mortality. Whereas mice treated with SiO_2_‐Tob microparticles had a 50% mortality at 168 h, Mg motors and Mg‐Tob microparticles groups both yielded a 62.5% survival on day 7. All of them were lower than CLP‐PBS group (mortality 100% on day 3). Notably, only one mouse treated with Mg‐Tob motors died until the termination of the study, which exhibited a significantly increased survival rate compared with other four groups. The dysregulated host response is caused by bacterial infection, which manifests itself in overwhelming inflammation firstly. Therefore, we believe that controlling infection is crucial to prevent the development of inflammation in sepsis. However, drug resistance may compromise the antibiotic effect in the progress of sepsis treatment, thus leading to high mortality of the SiO_2_‐Tob microparticles group in later stage. As for Mg motors, because of the powerful anti‐inflammatory effect of generated H_2_, the rise of mortality was prevented, but it still failed to achieve satisfactory effect with an around 40% mortality. We guess that it may be attributed to weak antibacterial activity of chitosan. The above survival analysis suggested that synergistic therapy of scavenging both infection and hyperinflammation is crucial to improve survival in sepsis. In the meantime, after CLP, the mice were monitored every 24 h within 7 d to record the body weight changes (Figure S9) and clinical scores according to a previously established method.^[^ ^28^ ^]^ The clinical scores were consistent with the survival results. CLP group with the highest score showed a number of typical signs of sepsis involving piloerection, huddling, severe diarrhea, decreased movement and listless appearance, indicating the successful establishment of sepsis model. As expected, the lowest score was observed for Mg‐Tob motors‐treated group, lower than that of Mg motors, SiO_2_‐Tob as well as Mg‐Tob microparticles (Figure 5c).

Sepsis is normally characterized by dysregulated systemic inflammatory response which leads to excessive secretion of pro‐inflammatory cytokines. Another batch of male BALB/c mice (6‐8 weeks old) were used to verify the anti‐inflammatory activity of Mg‐Tob motors in vivo. At 24 h after CLP, plasma and peritoneal lavage fluid were collected to evaluate the levels of TNF‐α, IL‐6 and IL‐1β using ELISA kits. As shown in Figure 5d, e, these inflammatory cytokines significantly elevated in plasma and peritoneal fluid of mice conducted with CLP, indicating the advent of systemic inflammation. The individual treatments via Mg motors and SiO_2_‐Tob microparticles both exhibited a relatively weak inflammation scavenging capability. It is well‐known that antibiotic itself does not possess anti‐inflammatory activity, hence such results could be attributed to the relationship between bacterial infection and inflammation progress as mentioned above. Unsurprisingly, the combination therapy of Mg‐Tob motors significantly alleviated systemic inflammation both in plasma and peritoneal cavity. The concentrations of TNF‐α, IL‐6 and IL‐1β dropped to the lowest level compared with other four groups. In addition, no statistical difference was seen in the expression levels of inflammatory cytokines between the Mg‐Tob motors group and sham group, further supporting the powerful anti‐inflammatory activity as well as survival benefit of our motors. Noteworthily, although combination therapy has advantages, these advantages were not evident in the Mg‐Tob microparticles group, which had lower survival rate and higher levels of inflammation than that of Mg‐Tob motors. We believe that the active motion of motor could facilitate H_2_ penetration and retention at the lesion site, thereby enhancing its anti‐inflammatory effect and survival benefit.

### 2.6 Prevention of Multiple Organ Failure In Vivo

**Figure 6:**
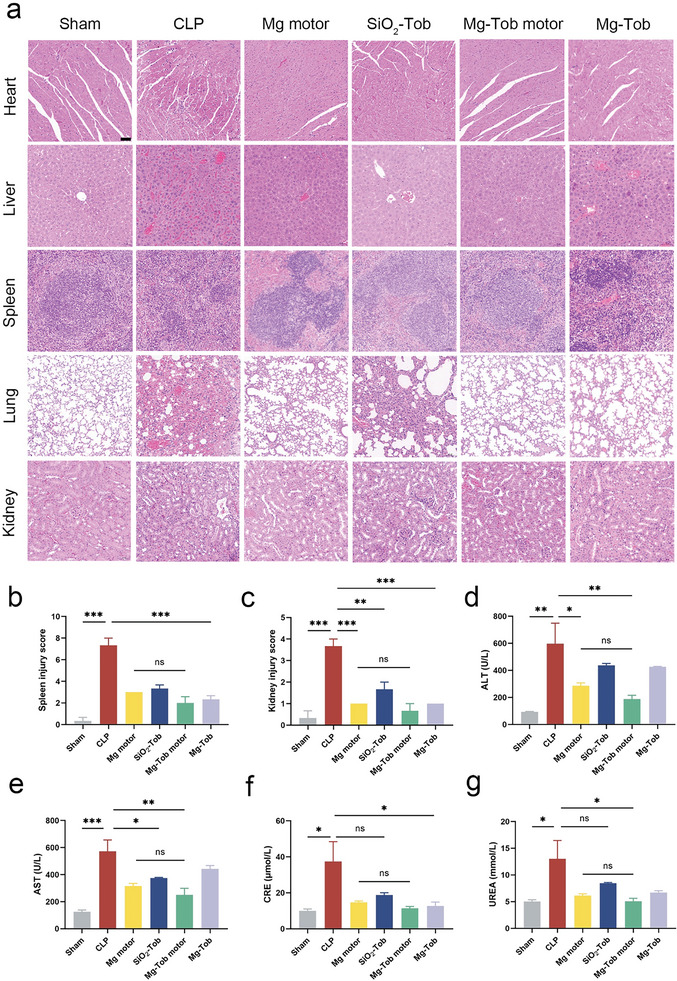
Prevention of multiple organ failure following CLP‐induced high‐grade sepsis. a) H&E staining of the collected heart, liver, spleen, lung and kidney. Scale bar: 50 µm. b) Spleen injury scores after different treatments. c) Kidney injury scores after different treatments. d‐g) Blood serum biochemistry parameters involving ALT, AST, CRE and UREA. Data represent mean ± SEM (n = 3), and the significant difference was analyzed by One‐way ANOVA with Tukey's post hoc test. *p < 0.05, **p < 0.01, ***p < 0.001 and ns representing non‐significance.

Apart from dysregulated systemic inflammation, high‐grade sepsis could lead to death owing to multiple organ failure. Histopathological and biochemical analyses were then carried out to determine the protective effect of our Mg‐Tob motors against multiple organ failure. Obvious pathological damages were observed in multiple organs involving spleen, lung and kidney of CLP mice. The histological spleen injury was scored based on the severity degree of necrosis, hemosiderin cells quantity and splenic nodules morphology. As shown in **Figure** **6**a, increased hemosiderin cells and focally aggregated small lymphocytes were observed in the spleen of CLP‐PBS group, indicating serious spleen dysfunction. Besides, the thickened alveolar walls, alveolar congestion and inflammatory cellular infiltration, distinguishing features of acute lung injury (ALI), were found in the lung.^[^ ^29^ ^]^ As for kidney, injured renal tubules and shrunken glomerulus were observed. It was worth mentioning that the individual antibiotic treatment via SiO_2_‐Tob microparticles exhibited severe multiple organ damage especially embodying in increased inflammatory cellular infiltration, while Mg motors treatment lightened the organ injuries to some extent by mitigating overwhelming inflammation. As expected, the lowest histopathological injury scores in spleen and kidney in Mg‐Tob motors group according to previously constructed criteria ^[^ ^30^ ^]^ were shown in Figure 6b,c, which reconfirmed that the synergistic therapy played a crucial role in improving multiple organ damage. Consistent with these improvements in organ histology, the serum levels of ALT, AST, CRE and UREA were also significantly down‐regulated in the Mg‐Tob motors group compared with the CLP‐PBS group (Figure 6d–g). Significantly, the efficacy of Mg‐Tob microparticles in the identical combination therapy was found to be inferior to that of the motors, thereby further reinforcing the benefits of motility in actively delivering hydrogen and Tob. In addition, the biocompatibility of Mg‐Tob motors was also investigated by histological analysis. After intraperitoneal injection of Mg‐Tob motors at the same dosage in healthy mice, heart, liver, spleen, lung and kidney were harvested and stained with Hematoxylin and eosin (H&E) after 24 h (Figure S10). No apparent pathological changes were observed in these organs when compared to the sham mice displayed above, thus suggesting the intraperitoneal administration of Mg‐Tob motors is deemed safe in vivo.

### 2.7 Antibacterial Performance of Mg‐Tob motors In Vivo

**Figure 7:**
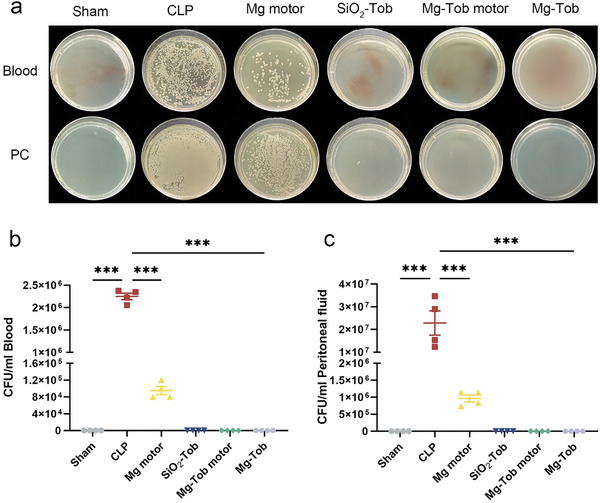
Antibacterial performance of Mg‐Tob motors in vivo. a) Bacterial colony photos. b‐c) CFU from blood and PC of mice with different treatments at 24 h post‐CLP. Data represent mean ± SEM (n = 4), and the significant difference was analyzed by One‐way ANOVA with Tukey's post hoc test. *p < 0.05, **p < 0.01, ***p < 0.001 and ns representing non‐significance.

Bacterial infection also plays a critical role in sepsis progression.^[^ ^31^ ^]^ Therefore, the blood and peritoneal lavage fluid of mice were collected at 24 h post‐CLP, and then bacterial colony forming units (CFU) in both of them were photographed and measured (**Figure** **7**a–c). CLP mice displayed a dramatically increased quantity of bacteria both in the peritoneal cavity (PC) and blood, indicating the progress from local to systemic infection. Whereas the Mg motors group exhibited weak antimicrobial activity, SiO_2_‐Tob microparticles, Mg‐Tob motors and Mg‐Tob microparticles containing the same concentration of Tob killed almost all of bacteria, demonstrating the necessity of combining with antibiotic to completely control bacterial infection.

## 3 Conclusion

In summary, we developed a biodegradable tobramycin‐loaded magnesium micromotor as a potential H_2_ generator and active antibiotic deliverer to simultaneously control dysregulated systemic inflammation and infection following sepsis. Compared with passive drug carriers, the water‐powered Mg‐based micromotor can move autonomously in peritoneal fluid, thus achieving active H_2_ and drug delivery for enhanced inflammation and infection scavenging. The Mg‐Tob motor conveyed satisfactory anti‐inflammation protection by neutralizing multiple pro‐inflammatory cytokines involving TNF‐α, IL‐6 and IL‐1β both in vitro and in vivo, which ensured it as an excellent candidate to prevent the development of highly heterogeneous inflammation in sepsis. By combining with antibiotic tobramycin, powerful antibacterial performance in vivo was achieved as well. Notably, the flexible multilayer structure of Mg‐based motor could be freely engineered for efficient and selective drug loading. In addition, the synergistic of our Mg‐Tob motor led to reduced multiple organ damage as well as significantly improved survival rate in a high‐grade sepsis model with no survival. Hence, we envision that the biocompatible micromotor could be served as an innovative and translational drug delivery system to realize a synergistic hydrogen‐chemical therapy against sepsis or other inflammation‐related diseases in the near future.

## 4 Experimental Section

### Materials

Mg microparticles (average diameter: 20 µm) were purchased from TangShan WeiHao Magnesium Powder Co., Ltd. Polylactic acid‐glycolic acid copolymer (PLGA, 8000 Da) was bought from Jinan Daigang Biomaterial Co., Ltd. Sodium alginate, chitosan and tobramycin were bought from Shanghai Macklin Biochemical Co., Ltd. Methylene blue (MB) and fluorescamine were purchased from Shanghai Aladdin Biochemical Technology Co., Ltd. DAPI was purchased from Beyotime Biotechnology. Recombinant Anti‐TNF‐α and Anti‐IL‐6 antibodies were purchased from Abcam. HyperScript III RT SuperMix for qPCR with gDNA Remover and 2×S6 Universal SYBR qPCR Mix were obtained from NovaBio. Cell Total RNA Isolation Kit was bought from Foregene Co., Ltd. ELISA kits for TNF‐α and IL‐6 were supplied by Dakewe Biotech Co., Ltd. IL‐1β ELISA kit was purchased from Elabscience (Wuhan, China). All other chemical regents used in this experiment were of analytical grade.

### Fabrication of Mg‐Tob motors and Mg‐Tob microparticles

Mg microparticles were firstly washed with isopropanol and acetone to remove the MgO passive layer, then dried under a N_2_ flow. Next, the obtained Mg microparticles were dispersed over a glass slide pre‐coated with PVP film, then 70 µL of PLGA (1 w/v%, ethyl acetate), 50 µL of Alg (0.1 w/v%, dissolved in 0.1% sodium dodecyl sulfate (SDS) aqueous solution.) containing the Tob (70 mg/mL) and 50 µL of Chi (0.1 w/v%, dissolved in slightly acidic aqueous solution) were sequentially coated onto Mg microparticles. After drying under vacuum, the resulting Mg‐Tob motors were scraped carefully from the glass slide. The same prewashing method was used to fabricate Mg‐Tob microparticles, which can gradually release hydrogen but without inducing motility. Firstly, PVP film was casted on a glass slide, followed by adding 50 µL of Alg. Subsequently, Mg microparticles were dispersed onto the thin layer of Alg, then another 50 µL of Alg containing Tob and 50 µL of Chi were sequentially coated onto Mg microparticles, so as to fabricate fully encapsulated and semipermeable Mg‐Tob microparticles.

### Characterization of Mg‐Tob motors

Mg‐Tob motors were stuck onto the conducting resin following by sputtering with gold using a SBC‐12 ion sputter coater (Beijing Zhongjingkeyi Technology Co., Ltd) before observation, then SEM image and element mapping were acquired by Phenom ProX with an accelerating voltage of 15 kV. Besides, to verify the multilayer structure of Mg‐Tob motor, PLGA layer and Alg‐Chi hydrogel were labeled with hydrophobic Nile red and hydrophilic FITC respectively during the preparation. The bright‐field and fluorescence images were captured by a Nikon Ti2‐A inversion fluorescence microscope.

### Tobramycin Quantification and Release from Mg‐Tob motors

The content of Tob was quantified based on fluorescence spectrophotometry. A series of standard Tob solutions (0‐2 µg/mL) were prepared to establish the standard curve. 2 mL of fluorescamine solution (dissolved in acetone, 0.15 mg/mL) was mixed with 2 mL of borate buffer (pH 8.5) and then diluted with deionized water containing tobramycin to form a 10 mL of Tob standard solution. 2 mL of each standard solution, after standing for 15 min, was extracted and detected by a fluorescence spectrometer with excitation and emission wavelength fixed at 388 nm and 469 nm. Mg‐Tob motors were dissolved in acid for 24 h before measurement. After centrifugation, supernatant was collected and diluted to the appropriate concentration. Next 0.4 mL of fluorescamine solution (0.15 mg/mL) and 1.5 mL borate buffer (pH 8.5) were added into 0.1 mL of the diluent sample solution. After standing for 5 min, the sample was tested by a fluorescence spectrometer and the loading amount of Tob was calculated according to the established calibration curve. For in vitro Tob release, Mg‐Tob motors (1 mg/mL) were added into a dialysis bag and subsequently incubated at 37°C under 80 rpm in the presence of PBS (pH 7.4). At specific time intervals, 1 mL of release medium was collected and an equivalent amount of PBS was replenished. The released Tob was quantified using the aforementioned method.

### Hydrogen Release from Mg‐Tob motors

The detection of H_2_ was based on the Redox reaction between MB and H_2_ under the catalysis of Pt nanoparticles. The former is able to form colorless reduced leucomethylene blue (leucoMB).^[^ ^32^ ^]^ The equation is as following:

(1) $$MB\left(blue\right)+2H^{+}+2e^{-}\to leucoMB\left(colorless\right)$$

Firstly, a series of standard MB solutions (1.875, 3.75, 7.5, 15, and 30 µM) were prepared and the absorbance of each 10 mL MB solution mixed with 10 µL Pt nanoparticles was tested by a UV spectrophotometer at 664 nm. Experimental groups including Mg‐Tob motors, Mg‐Tob particles and Mg microparticles (50 µg/mL) were added respectively into the MB‐Pt PBS solution (final volume of 2 mL). The concentration of H_2_ was calculated according to the absorbance difference of sample at 664 nm.

### Motion of Mg‐based motors

The motion of Mg motors and Mg‐Tob motors was evaluated in MPF added with 0.5 M NaHCO_3_ and 0.5% Triton‐X. A Nikon Ti2‐A inverted optical microscope equipped with a high‐speed camera and NIS Elements AR 3.2 software was used to record the motion within 20 s (time interval: 100 ms). The videos were analyzed by using Image J and Chemotaxis. At least 25 micromotors were tracked to calculate the accumulated distance and velocities.

### Cytotoxicity Evaluation

RAW 264.7 was used to investigate the cytotoxicity of Mg motors (0‐0.4 mg/mL), Mg‐Tob motors (0‐1 mg/mL) and SiO_2_‐Tob microparticles (0‐1.2 mg/mL). Cells were seeded and cultured for 24 h with 1 × 10^4^ cells per well in 96‐well plates, and the resulting cells were incubated with Mg motors, Mg‐Tob motors and SiO_2_‐Tob microparticles respectively for another 24 h. The viability of RAW 264.7 cells was quantified by CCK‐8 assay.

### Immunofluorescence Staining

Raw 264.7 cells were seeded and cultured for 24 h with 1 × 10^5^ cells per well in 12‐well plates, and the resulting cells were incubated with Mg motors (0.4 mg/mL), Mg‐Tob motors (1 mg/mL) and SiO_2_‐Tob microparticles (1.2 mg/mL) supplemented with LPS (1 µg/mL) respectively for 12 h. Cells with no treatment and solely treated with LPS (1 µg/mL) were considered as control groups. Then the cells were washed with PBS for three times and fixed in 4% paraformaldehyde for 15 min. To improve the permeability, the cells were treated with 0.1% Triton‐X for 10 min, and then blocked with 5% BSA for 1 h at room temperature. And then they were incubated overnight with monoclonal antibodies of TNF‐α and IL‐6 at 4°C, followed by PBS washing for 3 times and appropriate secondary antibodies incubation for 1 h at room temperature in dark. Finally, the cell nuclei were stained with DAPI. The corresponding images were taken by a Nikon Ti2‐A Inversion Fluorescence Microscope.

### Real‐Time PCR and ELISA Assays

RAW 264.7 cell were seeded and cultured for 24 h with 1 × 10^6^ cells per well in 6‐well plates, and the resulting cells were incubated with Mg motors (0.4 mg/mL), Mg‐Tob motors (1 mg/mL) and SiO_2_‐Tob microparticles (1.2 mg/mL) supplemented with LPS (1 µg/mL) respectively for 12 h. Cells with no treatment and solely treated with LPS (1 µg/mL) were considered as control groups. A Cell Total RNA Isolation Kit (Foregene) was used to isolate the total RNA, which was reverse‐transcribed into cDNA according to the standard protocols with a HyperScript III RT SuperMix for qPCR kit (NovaBio). Real‐time PCR was carried out with 2×S6 Universal SYBR qPCR (NovaBio) on a LightCycler 480 Instrument (Roche). PCR steps were described as following: Initial denaturation at 95°C for 10 min, denaturation at 95°C for 15 s and annealing/extension at 60°C for 1 min for 40 cycles. The relative expression of mRNA was quantified using the delta‐delta threshold cycle (Ct) method. After going through the same culture and administration procedures as above, the supernatants of the cells were collected to quantify the concentration of proinflammatory cytokines by using corresponding ELISA kits.

### CLP‐Induced High‐grade Sepsis Model

Male BALB/c mice, 6–8 weeks old, were provided by the Animal Experimental Center of Southern Medical University, Guangzhou, China. All animal experiments were approved by the Institutional Animal Care and Use Committee of Southern Medical University (SYXK 2016‐0041). CLP was performed according to the previous studies. Firstly, mice were anesthetized and shaved, then the peritoneal cavity was opened and the cecum was exposed carefully. 75% of cecum was then ligated tightly with a 3‐0 suture. After perforating the cecum with a 21‐gauge needle, a small drop of feces was squeezed out, then the cecum was placed back into the abdominal cavity. The peritoneum and skin were closed with corresponding surgical suture. Each mouse was resuscitated by injecting subcutaneously 1 mL of pre‐warmed saline. Mice from the sham group underwent the same laparotomy except for CLP. At 3 h, 12 h, 36 h after CLP, mice from different groups were injected intraperitoneally with PBS (200 µL), Mg motors (1 mg), Mg‐Tob motors (2.5 mg), Mg‐Tob microparticles (2.7 mg) and SiO_2_‐Tob microparticles (3 mg) respectively.

### Therapeutic Efficacy of Mg‐Tob motors In Vivo

Mice were monitored every 24 h within 7 d to record the survival rates and clinical scores according to a previously established method: score 0, no symptoms; score 1, piloerection and huddling; score 2, piloerection, huddling and diarrhea; score 3, lack of interest in surroundings and severe diarrhea; score 4, decreased movement and listless appearance; score 5, loss of self‐righting reflex. At 24 h post‐CLP, serial dilutions of blood and peritoneal lavage fluid were plated on the surface of a solid LB‐agar medium following by culturing at 37°C for 24 h. Then the plates were photographed and CFU were counted. Meanwhile, plasma and peritoneal lavage fluid were also used to quantify the concentrations of TNF‐α, IL‐6 and IL‐1β using ELISA kits. In addition, the serum levels of ALT, AST, CRE and UREA were tested using a biochemical analyzer. At 24 h after CLP, major organs including heart, liver, spleen, lung, kidney were harvested and stained with H&E.

### Statistical Analysis

Two‐sample comparisons were performed by the Student's t‐test and multiple comparisons were conducted by One‐way ANOVA with Tukey's post hoc test. GraphPad Prism 8.0 was used.

## Conflict of Interest

The authors declare no conflict of interest.

## Supporting information

Supporting Information

Supplemental Video 1

Supplemental Video 2

Supplemental Video 3

Supplemental Video 4

## Data Availability

The data that support the findings of this study are available from the corresponding author upon reasonable request.
