# Supplementary material for: Micromotor‐Enabled Active Hydrogen and Tobramycin Delivery for Synergistic Sepsis Therapy
Source: Adv Sci (Weinh). 2023 Oct 11;10(33):2303759. doi: 10.1002/advs.202303759 (PMC10667834; doi:10.1002/advs.202303759)
Supplement: Supplementary file 1 — Supporting Information [file ADVS-10-2303759-s005.pdf]

ADVANCED SCIENCE
Open Access

Supporting Information

for *Adv. Sci.*, DOI 10.1002/advs.202303759

Micromotor-Enabled Active Hydrogen and Tobramycin Delivery for Synergistic Sepsis Therapy

Yanzhen Song, Ruotian Zhang, Hanfeng Qin, Wenxin Xu, Jia Sun, Jiamiao Jiang, Yicheng Ye, Junbin Gao, Huaan Li, Weichang Huang, Kun Liu, Yunrui Hu, Fei Peng* and Yingfeng Tu**

Supporting Information

Micromotor-Enabled Active Hydrogen and Tobramycin Delivery for Synergistic Sepsis Therapy

*Yanzhen Song†, Ruotian Zhang†, Hanfeng Qin, Wenxin Xu, Jia Sun, Jiamiao Jiang, Yicheng Ye, Junbin Gao, Huaan Li, Weichang Huang, Kun Liu, Yunrui Hu, * Fei Peng, * and Yingfeng Tu**

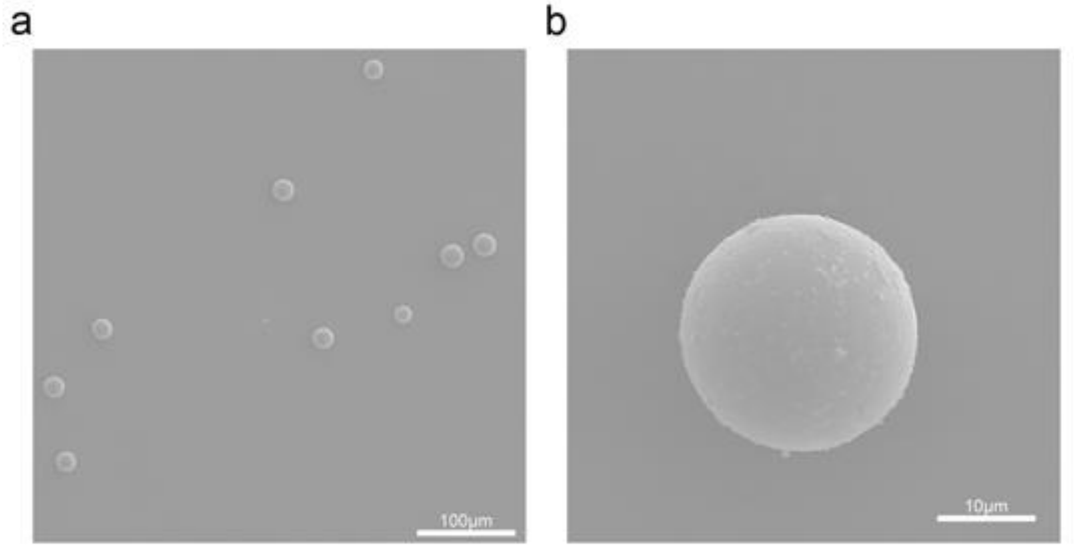

Figure S1. SEM images of Mg microparticles.

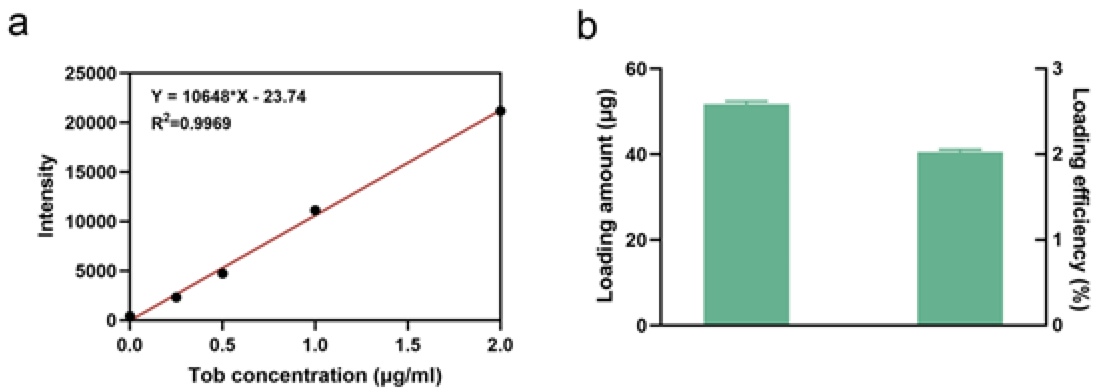

Figure S2. a) The calibration curve of tobramycin. b) The loading amount and loading efficiency of tobramycin in per 2.5 mg Mg-Tob motors.

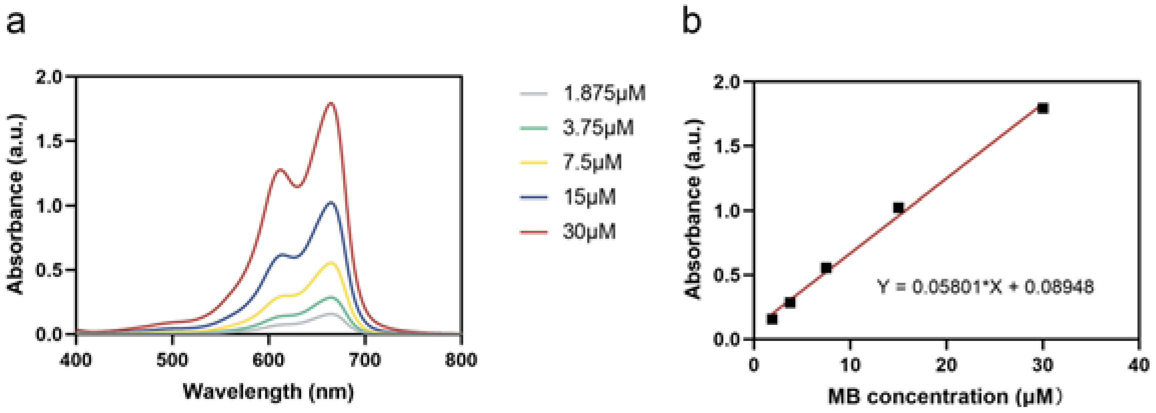

Figure S3. a) Absorption spectra of MB at a series of concentrations. b) The calibration curve of MB.

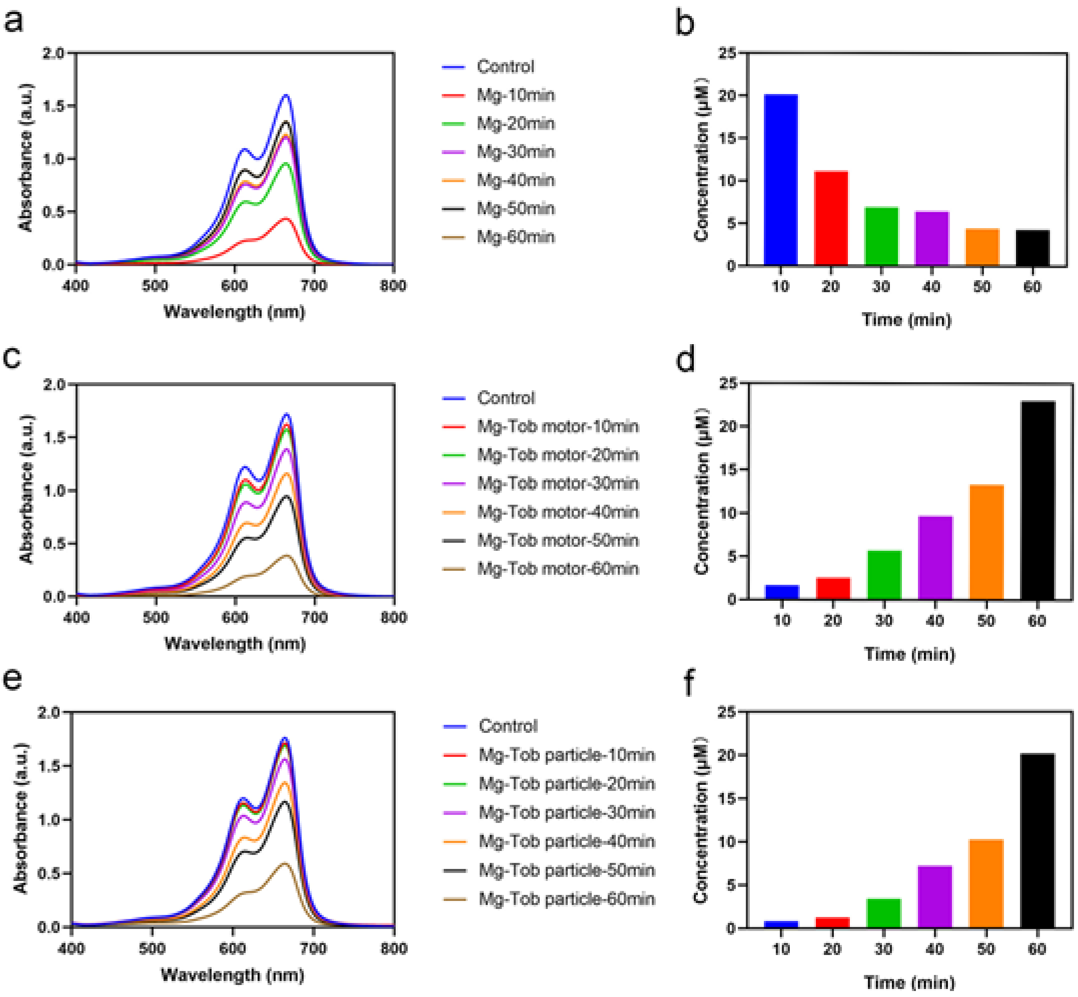

Figure S4. a) Absorption spectra of MB with Mg microparticles in PBS at different times. b) H_2 concentration generated by Mg microparticles in PBS at different times. c) Absorption spectra of MB with Mg-Tob motors in PBS at different times. d) H_2 concentration generated by Mg-Tob motors in PBS at different times. e) Absorption spectra of MB with Mg-Tob microparticles in PBS at different times. f) H_2 concentration generated by Mg-Tob microparticles in PBS at different times. The concentration of H_2 was calculated by the absorbance difference of sample at 664nm.

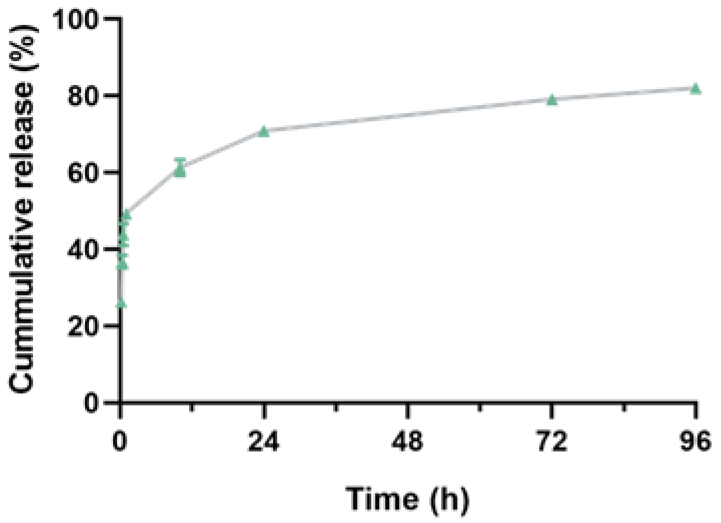

Figure S5. Tobramycin release profile from Mg-Tob motors at pH 7.4.

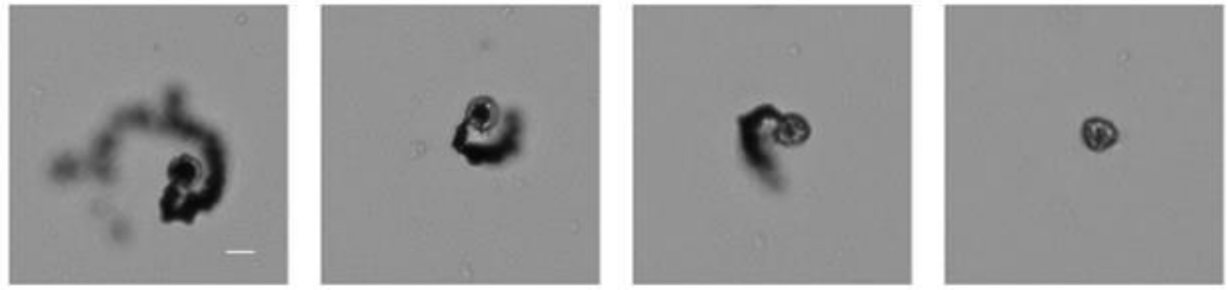

Figure S6. Images of single Mg-Tob motor and its left outer shell after the depletion of inner Mg microparticle. Scale bar: 20 μm.

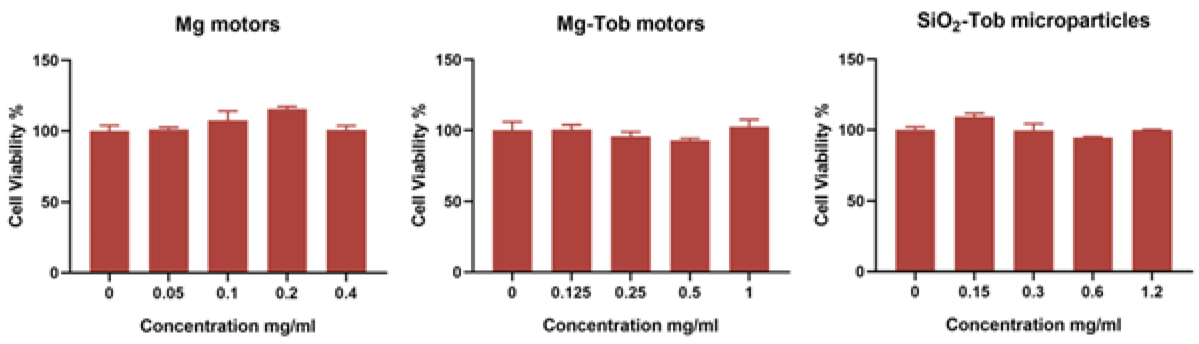

Figure S7. The viability of RAW 264.7 cells with different treatments for 24 h.

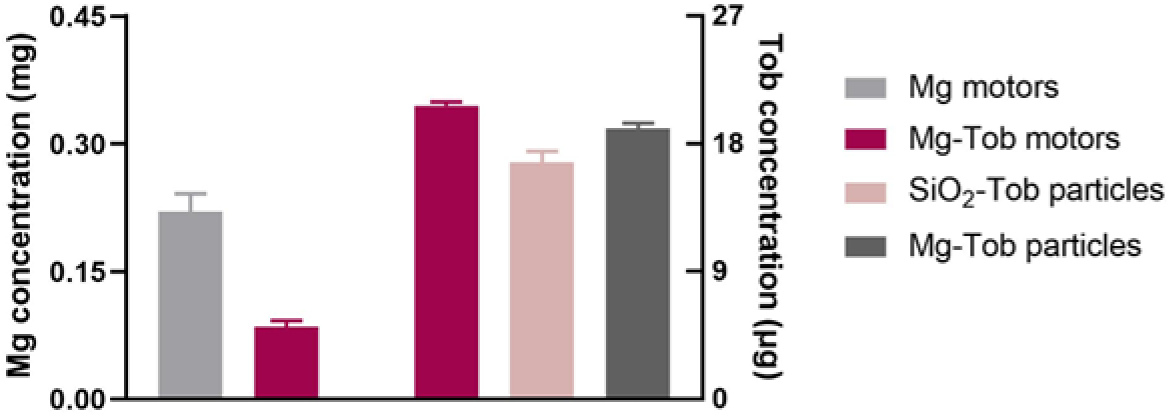

Figure S8. Quantification of magnesium in Mg motors and Mg-Tob motors (per 1 mg) by ICP-MS; the concentration of tobramycin in Mg-Tob motors, SiO_2-Tob microparticles and Mg-Tob microparticles (per 1 mg).

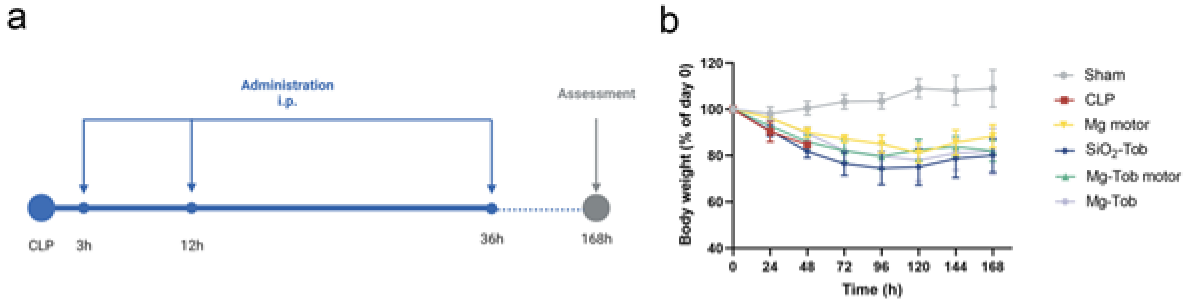

Figure S9. a) A therapeutic regimen for a CLP-induced high-grade sepsis model. b) Body weight changes of mice with different treatments after CLP within 168 h.

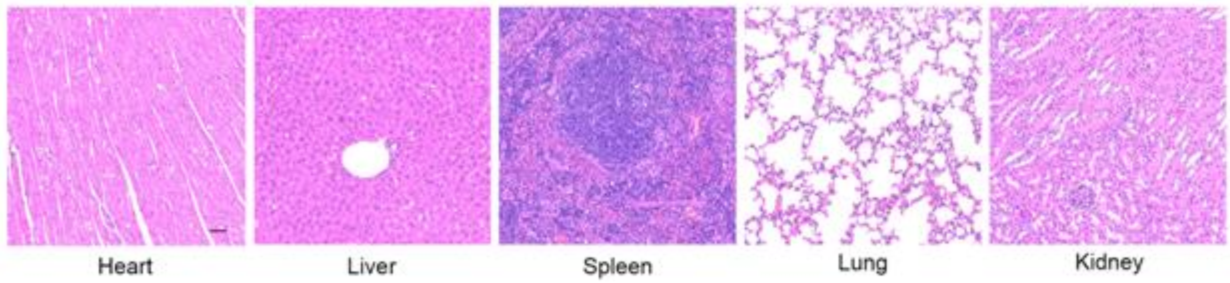

Figure S10. Representative H&E staining of the collected heart, liver, spleen, lung and kidney from healthy mice after intraperitoneal injection of Mg-Tob motors. Scale bar: 50 μm.
